# Can Quantitative Muscle Strength and Functional Motor Ability Differentiate the Influence of Age and Corticosteroids in Ambulatory Boys with Duchenne Muscular Dystrophy?

**DOI:** 10.1371/currents.md.1ced64dff945f8958221fddcd4ee60b0

**Published:** 2016-07-08

**Authors:** Cathleen Buckon, Susan Sienko, Anita Bagley, Mitell Sison-Williamson, Eileen Fowler, Loretta Staudt, Kent Heberer, Craig M. McDonald, Michael Sussman

**Affiliations:** Shriners Hospitals for Children-Portland, Portland, Oregon, USA; Shriners Hospitals for Children-Portland, Portland, Oregon, USA; Shriners Hospitals for Children-Northern California, Sacramento, California, USA; Research and Evaluation Section, California Department of Public Health, Shriners Hospitals for Children-Northern California, Sacramento, California, USA; UCLA Department of Orthopaedic Surgery, Kameron Gait and Motion Analysis Laboratory, Los Angeles, California, USA; UCLA Department of Orthopaedic Surgery, Los Angeles, California, USA; Department of Physical Medicine and Rehabilitation, University of California-Davis, Shriners Hospitals for Children-Northern California, Sacramento, California, USA; Shriners Hospitals for Children-Portland, Portland, OR, USA

## Abstract

Background: In the absence of a curative treatment for Duchenne Muscular Dystrophy (DMD), corticosteroid therapy (prednisone, deflazacort) has been adopted as the standard of care, as it slows the progression of muscle weakness and enables longer retention of functional mobility. The ongoing development of novel pharmacological agents that target the genetic defect underlying DMD offer hope for a significant alteration in disease progression; however, substantiation of therapeutic efficacy has proved challenging. Identifying functional outcomes sensitive to the early, subtle changes in muscle function has confounded clinical trials. Additionally, the alterations in disease progression secondary to corticosteroid therapy are not well described making it difficult to ascertain the benefits of novel agents, often taken concurrently with corticosteroids.

Objective: The purpose of this study was to examine outcome responsiveness to corticosteroid therapy and age at the onset of a natural history study of ambulatory boys with DMD.

Methods: Eighty-five ambulatory boys with DMD (mean age 93 mo, range 49 to 180 mo) were recruited into this study. Fifty participants were on corticosteroid therapy, while 33 were corticosteroid naïve at the baseline assessment. Within each treatment group boys were divided in two age groups, 4 to 7 years and 8 and greater years of age. The Biodex System 3 Pro isokinetic dynamometer was used to assess muscle strength. Motor skills were assessed using the upper two dimensions (standing/walking, running & jumping) of the Gross Motor Function Measure (GMFM 88) and Timed Motor Tests (TMTs) (10-meter run, sit to stand, supine to stand, climb 4-stairs). Two way analysis of variance and Pearson correlations were used for analysis.

Results: A main effect for age was seen in select lower extremity muscle groups (hip flexors, knee extensors and ankle dorsiflexors), standing dimension skills, and all TMTs with significantly greater weakness and loss of motor skill ability seen in the older age group regardless of treatment group. Interaction effects were seen for the walking, running, and jumping dimension of the GMFM with the naïve boys scoring higher in the younger group and boys on corticosteroid therapy scoring higher in the older group. The TMT of climb 4-stairs demonstrated a significant treatment effect with the boys on corticosteroid therapy climbing stairs faster than those who were naïve, regardless of age. Examination of individual items within the upper level GMFM dimensions revealed select motor skills are more informative of disease progression than others; indicating their potential to be sensitive indicators of alterations in disease progression and intervention efficacy. Analysis of the relationship between muscle group strength and motor skill performance revealed differences in use patterns in the corticosteroid versus naïve boys.

Conclusion: Significant muscle weakness is apparent in young boys with DMD regardless of corticosteroid treatment; however, older boys on corticosteroid therapy tend to have greater retention of muscle strength and motor skill ability than those who are naive. Quantification of muscle strength via isokinetic dynamometry is feasible and sensitive to the variable rates of disease progression in lower extremity muscle groups, but possibly most informative are the subtle changes in the performance characteristics of select motor skills. Further analysis of longitudinal data from this study will explore the influence of corticosteroid therapy on muscle strength and further clarify its impact on motor performance.

## Introduction

Duchenne muscular dystrophy (DMD), the most common neuromuscular disease of childhood, is characterized by a progressive myopathy that results in muscle wasting, weakness, and a loss of ambulation. In the absence of a curative treatment, corticosteroid therapy (prednisone, deflazacort) has been adopted as the standard of care, despite the adverse side effects seen in some individuals. Corticosteroids preserve cardiac and pulmonary function and enable the retention of motor skills and walking for an additional few years. 1 Age and disease progression are often considered when determining the optimal time to initiate corticosteroid therapy, resulting in variability in the age of onset and dosing regiments. 2 ^,^ 3

Recent advancements in the development of novel pharmacological agents that target the genetic defect underlying DMD, offer hope for a significant alteration in disease progression and ultimately lifespan. The primary focus of intervention in this population is the preservation of muscle function, and with therapeutic efficacy dependent upon muscle integrity at the onset, younger boys have been identified as the best candidates for clinical trials. However, the inherent heterogeneity of disease progression, the added confounder of normal maturation, and a limited repertoire of clinical outcome measures have challenged substantiation of intervention efficacy in this population. With the dramatic increase in the number of treatment agents in development for DMD and the need to differentiate the therapeutic efficacy of these therapies, the psychometric properties of clinical outcome measures have received greater scrutiny, and new disease specific outcome measures have been developed.^4^^,^ 5 Increasingly, evidence suggests that outcome measures targeting specific areas of interest or a carefully selected battery of outcomes are most effectively in identifying and differentiating therapeutic efficacy. 4

The purpose of this study was to characterize how corticosteroid therapy alters the natural history of boys with DMD using a battery of outcome measures selected to define patterns of muscle weakness, their relationship to function, and the features predictive of loss of ambulation in a large cohort of boys with DMD using a prospective multi-site study protocol. Since a number of the newer pharmacological agents are taken in addition to corticosteroids, it is important that we have a sound understanding of how disease progression in DMD has been altered by corticosteroids, within a multi-dimensional framework, in order to be able to determine the efficacy of newer agents. In this paper, a cross-sectional analysis of baseline assessments of quantitative muscle strength, functional motor skill ability, and timed motor tests were examined to determine measurement responsiveness to differences among age and treatment groups as well as the relationships among the outcome measures.

## Materials and Methods

Eighty-five ambulatory boys with DMD (mean age 93 mo, range 49 to 180 mo) were recruited into this multi-site study, thirty-two from Shriners Hospital for Children, Portland, OR, thirty-three from Shriners Hospital for Children, Sacramento, and twenty from University of California, Los Angeles, California from 2006-2014. Two boys were removed from analysis due to later reclassification as probable cases of Becker muscular dystrophy. Of the 83 participants, 50 participants were on corticosteroid therapy (mean age 104 mo, range 53 to 180 mo), while 33 were corticosteroid naïve (mean age 77 mo, range 49 to 153 mo) (Table 1) at the baseline assessment. Participants were divided into two age groups, 4-7 and ≥ 8 years of age, within each treatment group (corticosteroid and naïve). Age groups were selected based on historical trend in the literature for the outcomes of interest to enable comparison of study results. Within the naïve group a significant number of boys were of a younger age than in the corticosteroid group (Table 1). All participants on corticosteroid therapy for ≥1 month were allocated to the corticosteroid group due to documentation of the early therapeutic effects of corticosteroid therapy on muscle tissue using MRI/MRS in boys with DMD.^6^ Boys on corticosteroid therapy had been on therapy a mean of 29.3 mo, range 1-150 months. Ten percent (n=5; ages 53, 74, 84, 90, 97 months) had been on corticosteroid therapy for less than 4 months. Whether boys were on corticosteroid therapy was dependent upon the cooperative decision of the local site physician caring for the patient and family. Study participation did not influence this decision-making process. All study participants/parents signed informed assents and consents approved by local Institutional Review Boards (Oregon Health Sciences University, University of California, Davis, and University of California, Los Angeles). Study inclusion criteria included: 1) a diagnosis of DMD as determined by clinical evaluation, family history, genetic testing, 2) male, 3) four years of age or older, 4) ability to walk independently at self-selected speed for 10 minutes, 5) ability to cognitively understand directions for testing procedures. Assessments were completed in one three-hour visit when feasible. If this schedule was too arduous for the child, assessments were performed over two days. All assessment protocols and procedures were standardized across centers. Efforts were made to hold assessing clinicians constant at each center.

**Figure:**
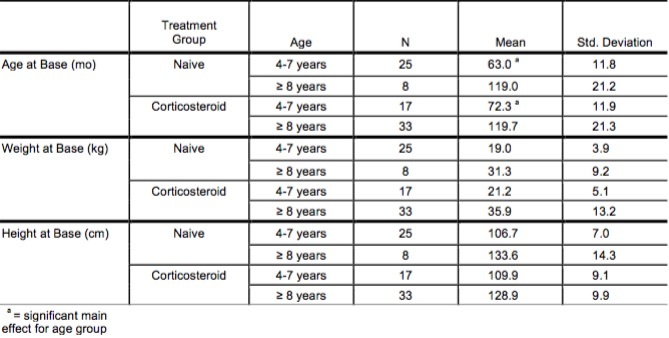
**Table 1: Descriptive Statistics**

## Measurements

**Volitional Muscle Strength**

Volitional muscle strength was assessed using the Biodex System 3 Pro isokinetic dynamometer. Select muscle groups were assessed unilaterally (based on hand dominance) as prior investigation has reported comparable strength bilaterally in boys with DMD.^7^^,^ 8 Isometric hip flexor strength was assessed in supine at 45° hip flexion, while extensor strength was assessed at 85° hip flexion. The Biodex pediatric knee attachment was used for assessing strength about the knee. Isometric knee flexor strength was assessed in sitting at 30° knee flexion, while extensor strength was assessed at 90° knee flexion. The isometric protocol about the hip and knee consisted of three five-second contractions performed consecutively by each muscle group with 10 second rests between contractions. Isokinetic concentric knee flexor and extensor strength was assessed at 60 °/sec, with the subject completing three consecutive arcs of knee extension and flexion through their full volitional range. Isometric ankle dorsiflexion and plantarflexion were assessed in semi-reclined sitting with the ankle in 0-5° of plantarflexion. The isometric protocol about the ankle consisted of three five-second contractions, alternating plantarflexion and dorsiflexion, with 10 second rest between each contraction. The order of muscle strength testing was held constant with strength about the ankle assessed first, followed by knee, and lastly the hip. The peak torque output for each muscle group and contraction type was used in the analysis. All torque values (Nm) were normalized by body weight (kg).

**Functional Motor Skills**

The skills included in the Standing (13 items) and Walk/Run/Jump (24 items) dimensions of the Gross Motor Function Measure (GMFM-88) were used in this study to assess motor skill ability in boys with DMD. While the GMFM has gained acceptance for its use in children with cerebral palsy and traumatic brain injury (TBI), it was originally developed in response to the paucity of measures in the field of developmental disabilities that demonstrated responsiveness to change in gross motor function over time. The gross motor skills included in the GMFM are based on normal development to capture the natural influence of maturation on motor skill development and the resultant alterations in skill acquisition due to motor deficits.^9^ All skills are typically mastered by five years of age. The GMFM has been shown to be valid, reliable and responsive in children with Down syndrome and spinal muscular atrophy, thus it is reasonable to consider the GMFM as an indicator of changes in muscle function in DMD.^9^^,^ 10 ^,^ 11 ^,^ 12 Although delayed, boys with DMD can demonstrate maturational motor skill acquisition in their younger years, prior to motor skill plateau and the subsequent onset of regression due to progressive muscle weakness; therefore, motor skill assessment can be informative. The GMFM is presently listed on the National Institute of Neurological Disorders and Stroke (NINDS), Common Data Elements (CDE) site (commondataelements.ninds.nih.gov) as a recommended instrument for use in DMD. In this study dimension point score sums were used in the analysis.

**Timed Motor Tests**

Four timed motor tests (TMTs) were assessed in this study including; assuming standing from supine on the floor, assuming standing from sitting on a bench (hips and knees at 90 degrees), climbing four standard stairs with assist of a railing, and ten-meter run on a level surface. Subjects were instructed to complete all measures as quickly as possible and were timed in seconds.

**Analysis**

Two-way analysis of variance (ANOVA), with treatment group (corticosteroid/naïve) and age (4-7 years, ≥ 8 years), was used to determine whether significant differences in the outcome variables of interest were revealed for the main effects of treatment and age, and whether interaction effects were present. Pearson product moment correlations were used to examine the relationships between outcome variables. Significance was set at p=.05.

## Results

**Volitional Muscle Strength**

No significant main effect for treatment group (corticosteroid therapy versus naïve) was seen in muscle strength; however, a main effect for age (4-7 years versus ≥ 8 years) was seen in select muscle groups. Isometric hip flexor strength (p=.037)(Figure 1), isometric and isokinetic concentric knee extensor strength (p=.017, p=.001)(Figures 2-3) and isometric ankle dorsiflexor strength (p=.024)(Figure 4) all demonstrated a significant decrement with age regardless of treatment group. No significant interaction effects were seen.

**Hip isometric muscle strength (group means):**
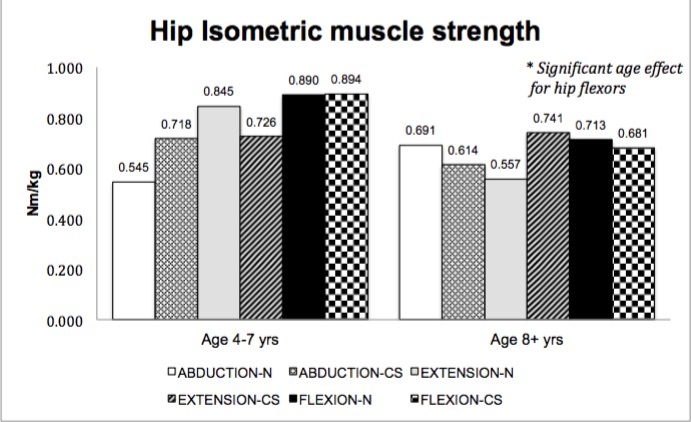

**Knee isometric strength (group means):**
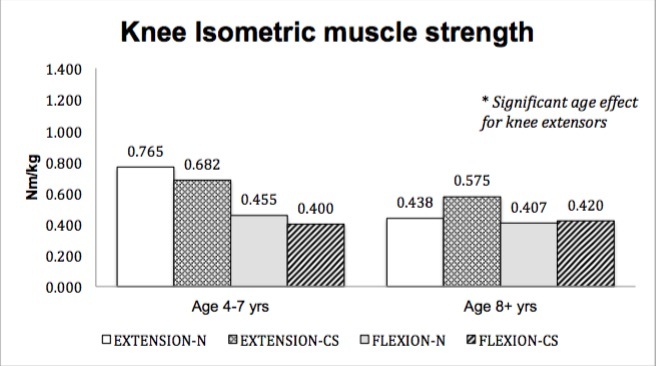

**Knee isokinetic concentric muscle strength (group means):**
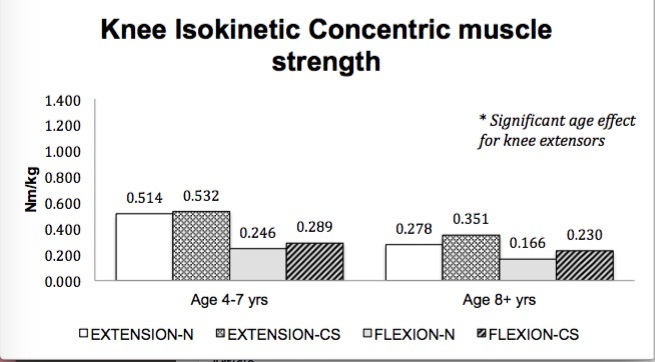

**Ankle isometric muscle strength (group means):**
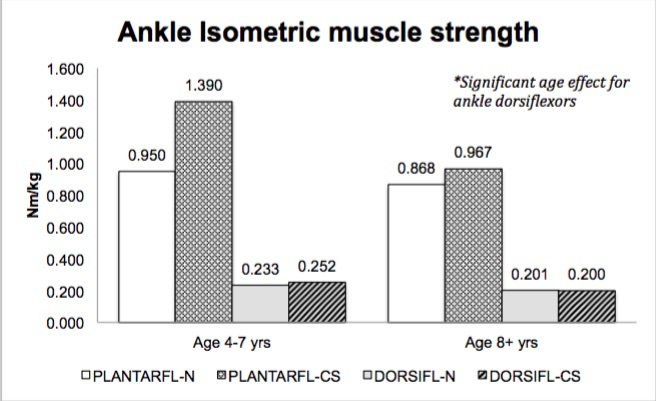

**Functional Motor Skills**

No significant main effect for treatment group (corticosteroid therapy versus naïve) was found in Standing dimension skills; however, a main effect for age (p=.011) was seen with lower standing scores in the older age group. A significant interaction effect was seen for Walking/Running/Jumping dimension skills (p=.046), with scores reversing across age. The higher scores in the naïve group when 4-7 years of age indicates a less advanced disease process and probably delay in initiation of corticosteroid therapy; conversely, the lower scores in the naïve group when ≥ 8 years may indicate greater severity of disease effect without the influence of corticosteroid therapy (Figure 5). In an attempt to identify the motor skills that are potentially more sensitive to incremental changes in muscle strength each individual Standing and Walking/Running/Jumping skill was analyzed.

**GMFM Standing and Walking, Running, & Jumping dimensions (group means):**
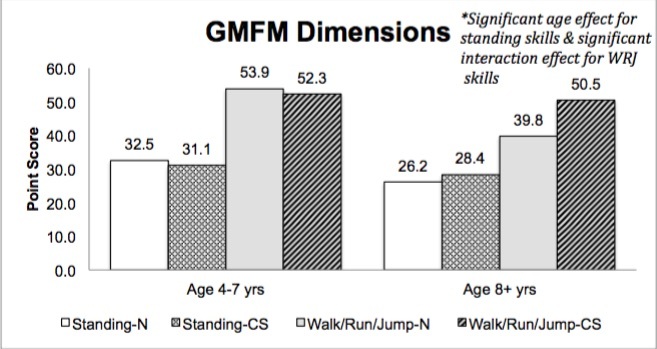

*Standing Skills*

Of the standing skills, six were not discriminatory in this study due to the inclusion criteria of this study (able to walk 10 minutes). The seven Standing skills that involved position transition demonstrated a significant main effect for age, with lower scores seen in the older age groups (Table 2). A significant interaction effect was seen for the skill of ‘lowering to the floor with control’ (p=.004) as the naïve group scored higher than the corticosteroid group when 4-7 years of age; however, the corticosteroid group demonstrated greater ability when ≥ 8 years.

**Figure:**
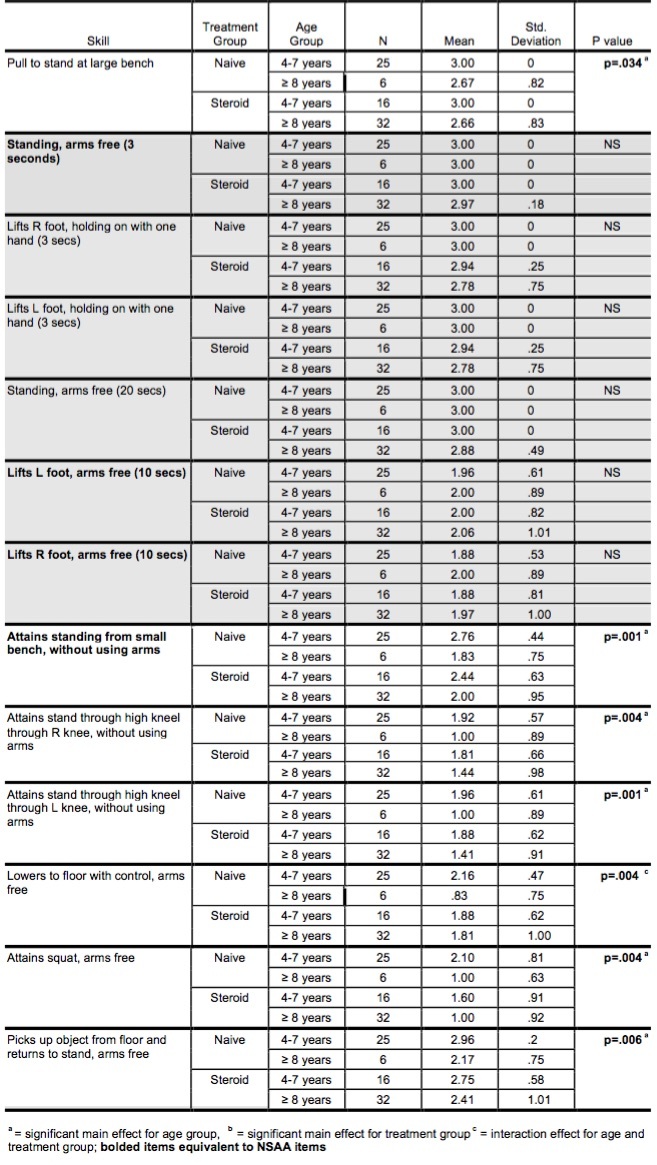
**Table 2: GMFM Standing dimension skill items (group means, standard deviation, p-values)**

*Walking, Running & Jumping Skills*

Similar to the finding that simple stance skills were not discriminatory, simple walking and running skills did not differentiate among age or treatment groups. Complex ambulatory skills (walking while carrying a large object with both arms, walking within an 8-inch pathway, climbing stairs with use of a railing) and jumping skills (jumping ‘up’ or ‘forward’ with two feet simultaneously) did demonstrate a main effect for age with skill decrement across age (Table 3). An interaction effect was seen for obstacle navigation while walking (stepping over a stick (p=.01) and jumping down (jumps off 6 inch step with both feet simultaneously (p=.036), as the naïve group scored higher than the corticosteroid group when 4-7 years of age; however, the corticosteroid group demonstrated greater ability when ≥ 8 years. ‘Hopping on one foot’ and ‘walks up 4 steps/walks down 4 steps alternating feet’ (without use of a railing), the most difficult skills determined by Rasch analysis of GMFM, did not demonstrate a main effect for age or treatment group. This finding suggests these skills may be informative early indicators of muscle strength in boys with DMD as only 20-30% of the steroid naïve group and 30-40% of the corticosteroid group were able to perform these skills.

**Figure:**
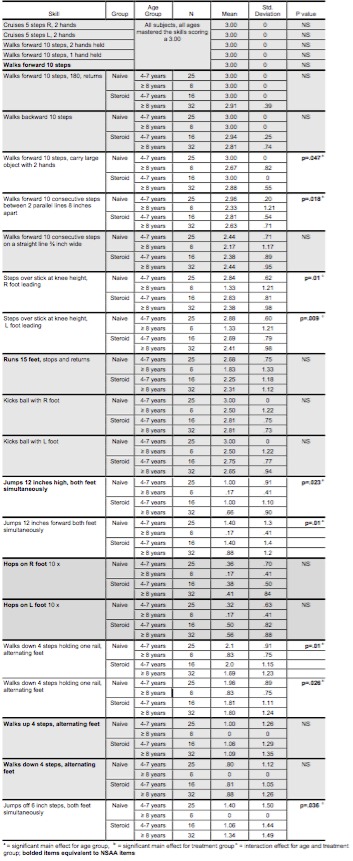
**Table 3: GMFM Walking, Running, & Jumping dimension skill items (group means, standard deviation, p-values)**

*Relationship between Volitional Strength and Functional Motor Skills*

Analysis of the relationships between lower extremity muscle strength and functional motor skills revealed significant fair (.25-.50) to moderate (.50-.75)^13^ positive correlations indicating that greater muscle strength was related to a greater ability to perform functional motor skills (Tables 4, 5). Significant relationships between muscle group strength and functional skills were more prevalent in the corticosteroid group than the naïve group. Standing skills correlated with hip extensor/flexor and knee extensor (isometric and isokinetic concentric) strength for corticosteroid group, while only hip and knee extensor (isokinetic concentric) strength were significant for the naïve group (Table 4, 5). During Walking, Running & Jumping skills both the extensor/flexor strength about the hip and knee correlated significantly for the corticosteroid group, while only knee extensor strength (isometric and isokinetic concentric) correlated for the naïve group.

**Figure:**
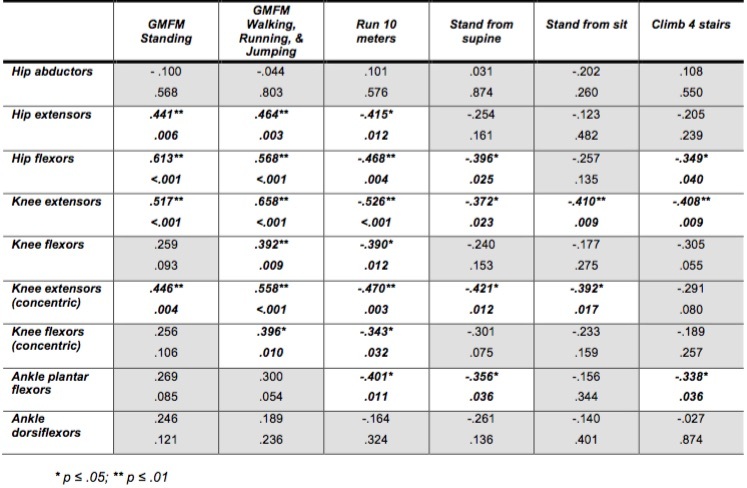
**Table 4: Corticosteroid group: Correlations for muscle strength, functional motor skills and TMTs (Pearson r and p-values)**

**Figure:**
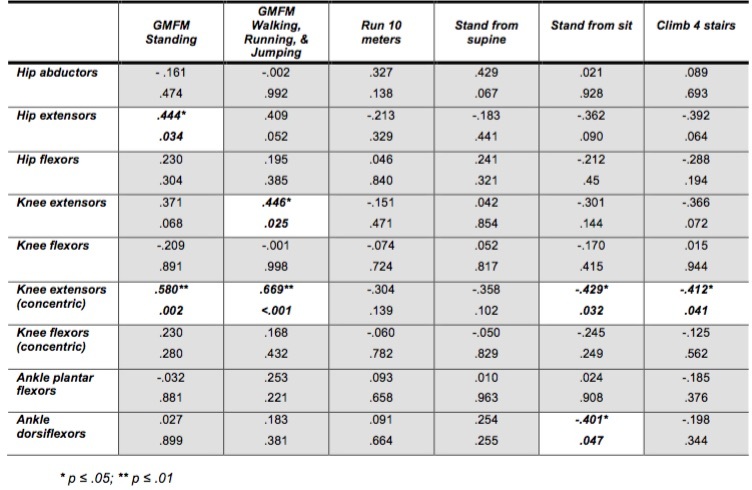
**Table 5: Corticosteroid Naïve: Correlations for muscle strength, functional motor skills and TMTs (Pearson r and p values)**

**Timed Motor Tests**

The only TMT to demonstrate a main effect for treatment was ‘climb 4 stairs’ (p=.042) as the corticosteroid group demonstrated significantly greater ability at > 8 years of age than the naïve group. A main effect for age was found for all TMTs; ‘run 10 meters’ p=.043, ‘supine to stand’ p=.026, ‘sit to stand’ p=.001, and ‘climb 4 stairs’ p=.002, with the older age group taking significantly longer to perform each TMT (Figure 6).

**Timed Motor Tests (group means):**
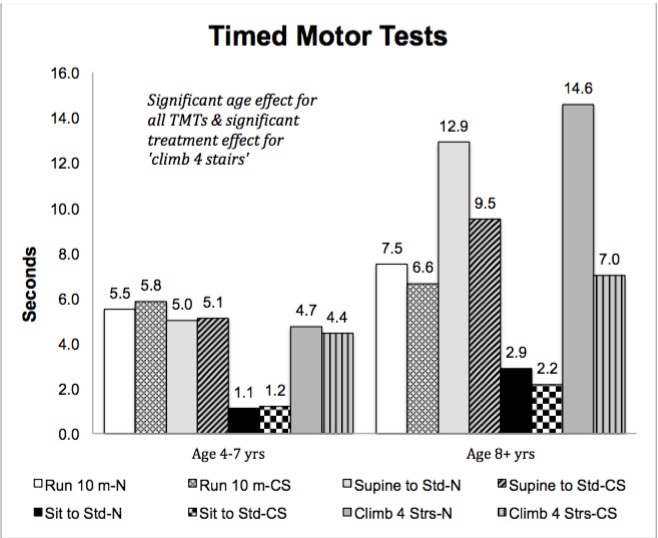

*Relationship between Volitional Strength and Timed Motor Tests*

As in the functional motor skills, significant relationships between muscle group strength and TMT were more prevalent in the corticosteroid group than the naïve group. All correlations between muscle strength and TMT were negative indicating that as muscle strength decreased the time to perform the TMT increased. In the corticosteroid group ‘run 10 meters’ demonstrated a significant negative correlation with force production by hip and knee extensors/flexors and ankle plantar flexors; ‘stand from supine’ and ‘climb 4 stairs’ demonstrated a significant negative correlation with strength of hip flexors, knee extensors, and ankle plantar flexors; and ‘stand from sit’ correlated only with knee extensors (Table 4). The naïve group demonstrated significant correlations between force produced by knee extensors (isokinetic concentric) and ankle dorsiflexors and ‘sit to stand’, while only knee extensors (isokinetic concentric) correlated with ‘climb 4 stairs’ (Table 5).

## Discussion

**Volitional Muscle Strength**

While quantitative methods of measuring strength have become more prevalent, they remain limited, and thus the relationship between the muscle group strength and functional mobility remains difficult to ascertain. Reliable methods of monitoring alterations in muscle strength are vital to understanding the progression of DMD, albeit, volitional strength measurement is dependent upon the child’s ability to reliably produce maximal efforts. Hand held dynamometers (HHD) are common in the clinical environment due to their low cost, portability, and universality; however, isokinetic dynamometers (ie Biodex System) provide a more psychometrically sound assessment of volitional strength as well as real-time visual feedback during force production, which assists in motivating and reinforcing maximal effort in children. Presently, studies that have used isokinetic dynamometers for strength assessment in DMD boys are limited, thus the sensitivity and specificity of the method of quantitative measurement has not been established and standardized in this population; however, emerging evidence is encouraging.^14^

In a natural history study McDonald et al (1995) assessed quantitative muscle strength about the knee using isokinetic dynamometry in boys with DMD 6-12 years of age (treatment regime unspecified) and age-matched controls. By six years of age boys with DMD demonstrated an isometric knee extensor/flexor strength of 50%/32% of controls, and an isokinetic concentric knee extensor/flexor strength of 22%/28%, respectively. A relatively linear decrement in strength from 6-12 years of age, with the rate of decline in greater in extensor than flexor muscle groups, was seen in DMD boys.^7^^,^ 15 A recent analysis of strength using isokinetic dynamometry in boys with DMD of similar age (5.6-12 years of age) (71% on corticosteroid therapy) reported isometric knee extensor/flexor strength to be 18%/40% of controls, and isokinetic concentric knee extensor/flexor strength to be 21%/39%. Longitudinal analysis revealed boys with DMD had an increase in isometric and isokinetic concentric knee extensor/flexor strength when <7.5 years of age; conversely, decrements were seen in these muscles in the boys over 7.5 years of age, with greater decline after 9 years of age.^14^ While the variation in strength reported in boys with DMD in the studies above could be attributed to the heterogeneous nature of DMD or differences in treatment regimes, it should be noted that variability in muscle strength is typical in normal development. With normally developing children 4-12 years of age strength variability is high within each age group and increases with age.^14^^,^ 15 The notable finding in boys with DMD is that muscle weakness is significant early in the disease process, and despite the reported gains in strength in boys with DMD <7.5 years, their strength profile diverges from that of normal children 4-12 years of age who demonstrate a gradual and almost linear increase in muscle strength, regardless of gender, until the onset of puberty.

This study supports the above noted studies in that significant weakness was seen in the younger boys (4-7 years) relative to age-matched norms, and the decline in muscle strength across age was significant. Comparison of isometric and isokinetic concentric knee extensor/flexor strength of DMD boys, 4-7 years of age, in this study with a normative age-matched data^16^^,^ 17 revealed that the corticosteroid group had 45%/48% of isometric and 39%/36% of isokinetic concentric knee extensor/flexor strength of controls, respectively; while the corticosteroid naïve group had 43%/41% of isometric and 38%/30% of isokinetic concentric knee extensor/flexor strength. At ≥ 8 years of age the corticosteroid group demonstrated 17%/18% of isometric and 19%/21% of isokinetic concentric knee extensor/flexor strength of controls, respectively, while the naïve group had 14%/13% of isometric and 15%/15% of isokinetic concentric knee extensor/flexor of controls. Although significant differences in the strength of the corticosteroid and naïve groups were not found in this study, the boys on corticosteroid therapy did tend to be slightly stronger. Recent examination of the impact of corticosteroid therapy on muscle tissue using magnetic resonance imaging (MRI) and magnetic resonance spectroscopy (MRS) in combination with isokinetic dynamometry^6^ in boys with DMD (5-6.9 years of age) revealed a suppression of muscle inflammation and fatty tissue infiltration in the muscles studied. While tissue changes were associated with significantly greater isometric knee extensor strength in the corticosteroid group than an age-matched naïve group, tissue changes in the soleus were not associated with significant strength differences, indicating the need for further investigation of the relationship between tissue changes and volitional strength.

While isokinetic dynamometry has been shown to be a sensitive and responsive method of measuring muscle strength in boys with DMD^14^ standardization of testing procedures (i.e. data normalization, testing positions and velocities) is needed to allow for valid comparison of results across studies. Future studies combining the use of isokinetic dynamometry, MRI, and select functional outcomes have the potential to greatly enhance our understanding of the primary and compensatory changes in muscle function characteristic of DMD and to expedite the identification of therapeutic agents that alter muscle integrity and function.

**Functional Motor Skills**

Boys with DMD are known to lose functional motor skill capability with progressive muscle deterioration and ultimately loss of ambulation; however, the pattern and rate of skill loss is variable. Methodological review of the DMD literature has suggested that motor scales that assess a wide range of abilities may be more informative in characterizing different phases of the disease process, whereas disease-specific motor tests might be more appropriate for short term efficacy studies.^18^ Due to the functional ability of the boys at the onset of this study and the interest in the exploring ambulatory skill performance, only the Standing and Walking, Running & Jumping skill dimensions of the GMFM-88 were used. The preliminary findings of this study demonstrate that these dimensions were sensitive to changes in gross motor function across age in boys with DMD; additionally, the Walking, Running, & Jumping dimension was able to discriminate differences in skill ability between the corticosteroid and corticosteroid naïve groups.

*Standing Skills*

In ambulatory boys with DMD skills that involved transitions in and out of standing were the items discriminatory across age in this ambulatory cohort, with greater skill loss seen in the older boys with more advanced disease. ‘Assuming stand through half-kneel’ and ‘lowering to the floor from standing’, which are not usually seen in disease-specific motor scales for DMD, appear to be informative indicators of disease progression and possibly therapeutic efficacy. Lowering to the floor from standing requires generation of a controlled eccentric contraction of the extensor muscles of the hip, knee and ankle, the greatest force a muscle can produce, to control the lowering of the center of mass. This skill was sensitive to age and treatment in this study, and thus may be a sensitive early indicator of disease progression and intervention efficacy.

*Walking, Running & Jumping Skills*

Simple walking activities were not discriminatory in this cohort, however, walking items that included additional tasks or constraints were better indicators of disease status and the possible mitigating influence of corticosteroid therapy. Prior investigation has reported that the progression of muscle damage in DMD is workload dependent, and thus can be seen first in movements of the whole body against gravity, with jumping and running (which require both feet to be off the ground simultaneously) being the first skills to fail.^19^ While running skills were less responsive to early disease progression in this cohort, jumping skills were already significantly impaired in the most of the boys, with hopping skills never attained in the majority of the boys in this study. Hopping skills normally emerge at three years of age, with most able-bodied children able to hop at least once. By five years of age mastery is achieved with hopping ten times on one foot easily attained. One legged hopping is considered to be the most advanced jumping skill and has been shown to be strongly related to age and thigh muscle strength^20^ , which is known to be affected early in boys with DMD, thus is a early indicator of gains or losses in strength. Descending and ascending 4 stairs (no rail), per Rasch analysis of the GMFM, is second in difficultly, preceded by one-legged hopping. Most of the boys with DMD in this study were unable to stair walk without rail use.

Since the onset of this study a variety of disease-specific functional scales that assess different ranges of motor abilities have been developed and validated for DMD, including the Motor Function Measure (MFM)^21^ and the North Star Ambulatory Assessment (NSAA),^5^^,^ 22 thus the repertoire of appropriate outcome measures for DMD is expanding. The NSAA was developed specifically to identify functional change and therapeutic efficacy in ambulatory boys with DMD and is used in a number of clinical trials.^5^^,^ 14 ^,^ 22 ^,^ 23 Eleven of the 17 NSAA skills are equivalent to skills assessed within the Standing and Walking, Running & Jumping Dimensions of the GMFM. Differences are seen between the scales in how the motor skill is graded with the GMFM stratifying responses over a 4-point scale, while the NSAA uses a 3-point scale. Further investigation of the various dimensions of the GMFM is needed to determine its sensitivity to change in this population; however, its skill range, specificity within dimension, and scoring methodology may be more discriminatory than other assessments and thus more likely to detect incremental change in motor skill performance in boys with DMD.^9^

**Timed Motor Tests**

Timed motor tests (TMTs) such as the 10 meter walk, rising from floor, rising from chair, and walking up 4 steps have become the standard approach to determining peak motor performance.^8^^,^ 24 ^,^ 25 The use of TMTs in boys with DMD has become an accepted method for motor function staging and predicting clinically meaningful endpoints in motor function, such as loss of ambulation, which is informative to patients and their families.^26^^,^ 27 ^,^ 28 The benefits of TMTs are proposed to be ease of use and responsiveness to change^29^^,^ 30 ^,^ 31 , and the ratio data generated that allows for parametric analysis; however, the reliability of TMTs has been questioned, as the potential for random error in some measures (stand from sit) is significant.^24^ In this study TMTs were sensitive to disease progression with age, but only the time to ‘climb 4 stairs’ indicated a difference in ability between the corticosteroid and naïve groups. Further study is indicated to determine if TMTs can reliably detect the early, subtle changes in muscle function induced by newly introduced therapeutic agents.

*Relationship between muscle strength and motor skills*

A relationship between muscle strength and functional motor skill ability is known in DMD, but not well understood. Muscle weakness in DMD has been reported to have a proximal to distal progression, with involvement of large proximal hip and shoulder girdle muscle noted first followed by distal extremities and the trunk.^32^ Various muscle groups have been reported to be predictive of ambulatory loss;^19^^,^ 33 however, recent studies of muscle tissue changes using MRI revealed considerable variation in disease progression across thigh muscles, and within select muscles within muscle groups.^19^^,^ 32 Thus, it is probable that the contribution of various muscle groups to the eventual loss of ambulatory skills may be more variable than appreciated within this population. A recent study of boys with Becker muscular dystrophy suggested that functional motor skills are muscle group dependent, and the patterns of change are reflective of a given disease progression, thus clinical study designs should select outcomes relevant to disease progression when evaluating intervention efficacy.^3^ In this study the relationships between muscle group strength and motor function varied based on the motor ability being measured and treatment group. While the steroid group demonstrated relationships between the strength of various muscle groups and functional motor skills (GMFM, TMTs), these relationships were dramatically reduced in the naïve group. Whether this finding is indicative of the various compensatory strategies employed as lower extremity weakness progresses, or other contributing factors warrant further investigation.

## Conclusion

The baseline data analysis of this natural history study indicates that the outcomes measures utilized in this study were sensitive to the age related differences in strength and motor function that are characteristic of disease progression boys with DMD; however treatment effects were less likely to be identified. These findings reflect the difficulty inherent in obtaining the statistical power needed to substantiate intervention efficacy in the small, heterogeneous samples sizes that are characteristic of DMD clinical studies. Isokinetic dynamometry revealed variability in the muscles affected, which has been corroborated with magnetic resonance imaging (MRI) of thigh muscle tissue,^32^ with significant muscle weakness present in lower extremity muscle groups at an early age. Most informative was the sensitivity of the GMFM dimensions to age and disease related changes in muscle strength and resultant motor skill performance in this cohort, specifically the select motor skills that offer the potential to be sensitive indicators of early disease progression, and thus probable indicators of intervention efficacy. The differences seen in the relationship between muscle group strength and motor skill performance for boys on corticosteroid therapy versus those who are naïve indicates further study is needed to determine the compensatory strategies (i.e. muscular, postural, etc.) employed to preserve functional ability with decrements in muscle strength. Analysis of the longitudinal data of this study will further delineate the psychometric attributes of these outcome measures and advance evidence of how corticosteroid therapy alters the natural history of disease progression in DMD.

## Competing Interests

We have read the journals policy and we have the following conflicts:

Susan Sienko, Research Associate: Shriners Hospitals for Children. “Biomechanical Analysis of Gait in Individuals with Duchenne Muscular Dystrophy” with Sussman, (PI), 1/06 – 12/15.

Cathleen E Buckon, Research Associate: Shriners Hospitals for Children. “Biomechanical Analysis of Gait in Individuals with Duchenne Muscular Dystrophy” with Sussman, (PI), 1/06 – 12/15.

Eileen Fowler, Local Principal Investigator (UCLA): Shriners Hospitals for Children. “Biomechanical Analysis of Gait in Individuals with Duchenne Muscular Dystrophy” with Sussman, (PI), 1/10 – 12/15.

Anita Bagley, Local Principal Investigator (NCal): Shriners Hospitals for Children. “Biomechanical Analysis of Gait in Individuals with Duchenne Muscular Dystrophy” with Sussman, (PI), 1/06 – 12/15.

Loretta Staudt, Research Associate (UCLA): Shriners Hospitals for Children. “Biomechanical Analysis of Gait in Individuals with Duchenne Muscular Dystrophy” with Sussman, (PI), 1/10 – 12/15.

Mitell Sison-Williamson, Research Associate (NCal): Shriners Hospitals for Children. “Biomechanical Analysis of Gait in Individuals with Duchenne Muscular Dystrophy” with Sussman, (PI), 1/06 – 12/15.

Kathy Zebracki: None.

Craig M McDonald, Local CoI: (NCal): Shriners Hospitals for Children. “Biomechanical Analysis of Gait in Individuals with Duchenne Muscular Dystrophy” with Sussman, (PI), 1/06 – 12/15.

Michael D Sussman, Principal Investigator: Shriners Hospitals for Children. “Biomechanical Analysis of Gait in Individuals with Duchenne Muscular Dystrophy”, 1/06 – 12/15.
